# Estimation of Cardiac Troponin-T Levels in Acute Myocardial Infarction With Reference to Short-Term Prognosis

**DOI:** 10.7759/cureus.92063

**Published:** 2025-09-11

**Authors:** Sai Venkatram Reddy Molugu, Chaitra Reddy Molugu, Venkata Bhargava Chalasani, Srinivasa Reddy Badvel

**Affiliations:** 1 Medicine, Osmania Medical College, Hyderabad, IND; 2 Medicine, Kamineni Academy of Medical Sciences and Research Centre, Hyderabad, IND; 3 General Medicine, Nimra Institute of Medical Sciences, Vijayawada, IND

**Keywords:** acute myocardial infarction, cardiac troponin t, heart failure, positive predictive value, recurrent angina

## Abstract

Aim: This study aimed to estimate the cardiac troponin-T (cTnT) levels in patients with acute myocardial infarction (AMI) to measure the short-term prognosis.

Method: Sixty patients with symptoms of AMI within six hours of onset were included, and the prognostic significance of cTnT levels was evaluated to predict mortality, recurrent ischemic events, and heart failure. The diagnostic performance of cTnT was evaluated through sensitivity and specificity analysis based on the determined cut-off value. All participants underwent clinical evaluations, echocardiograms, and received thrombolytic therapy.

Results: Among study participants, 45 patients were male (75%) and 15 were female (25%), and the mean age was 50.7±7.93 years. Cardiac troponin T was positive (cTnT >0.18 ng/mL) in 50 (83.34%) cases and negative in 10 (16.66%) cases. The mean cTnT levels were higher in patients who did not survive than those who did (2.92±1.43 vs 0.705±0.63 ng/mL). A cTnT levels between patients with and without recurrent angina shows a significant difference (1.9705±1.538 vs 0.82±0.915 ng/mL, p<0.001). A cTnT level of 0.22 ng/mL at presentation diagnosed AMI with a sensitivity of 76.92% and a negative predictive value (NPV) of 98.2%, and a specificity and positive predictive value (PPV) of 100% and 65.4%, respectively. A cut-off cTnT value of 2.2 ng/mL predicted mortality with a sensitivity of 76.92% and a negative predictive value (NPV) of 94%, and a specificity and positive predictive value (PPV) of 100% each. The multivariable analysis revealed several factors significantly associated with mortality: point-of-care cardiac troponin T ≥0.22 ng/mL (hazard ratio (HR): 1.95, 95% confidence interval (CI): 1.85-2.19), heart failure (HR: 1.85, 95% CI: 1.65-2.08), recurrent angina (HR: 1.38, 95% CI: 1.19-1.55), and reinfarction (HR: 1.04, 95% CI: 0.99-1.12).

Conclusion: Cardiac troponin T positivity at admission consistently correlated with decreased rates of reperfusion after thrombolysis and lower ejection fraction. Cardiac troponin-T levels at admission can be able to predict the short-term prognosis, mortality, recurrent ischemic events, and heart failure in patients with AMI.

## Introduction

Acute myocardial infarction (AMI) is a leading cause of death globally, with a mortality rate of approximately 15% [1]. AMI occurs due to a sudden cessation of coronary blood flow, creating an imbalance between the myocardium's oxygen demand and supply, which leads to ischemia and subsequent tissue necrosis [2,3]. The economic burden of AMI is substantial, with annual costs estimated at $84.9 billion, which includes direct medical expenses and lost productivity due to illness and premature death [4-6]. A 10-year study revealed that patients experiencing AMI are becoming older and presenting with a higher number of cardiovascular risk factors [7,8]. In North East India, tobacco usage and smoking are recognized as the most significant risk factors for AMI, along with other critical contributors such as sedentary lifestyles, high serum triglyceride levels, hypertension, and diabetes [9,10]. The healthcare system in India, which operates within a mixed economy, frequently leads to delayed presentations [11,12]. This delay reduces the chances of patients receiving evidence-based treatments, ultimately contributing to a higher 30-day mortality rate [13,14].

The administration of thrombolytic agents for pharmacological lysis has become a standard treatment approach for those suffering from acute myocardial infarction [15]. Cardiac troponin T (cTnT) is recognized as the most reliable biomarker for diagnosing ST-segment elevation myocardial infarction (STEMI). The cardiac troponin complex plays a crucial role in the contractile function of striated muscle. Acute myocardial injury results in the breakdown of troponin into its individual subunits: C, I, and T. Troponin T can be detected in the bloodstream within four to six hours after a myocardial infarction and can remain detectable for five to 14 days. A troponin T level of 0.1 ng/mL (0.1 µg/L) or higher is considered indicative of myocardial infarction. While there is evidence supporting the individual prognostic significance of this marker in patients with AMI, its evaluation has yet to be fully investigated [16,17]. The aim of this study was to evaluate cardiac troponin-T levels in patients who had suffered from AMI within six hours of symptom onset. Furthermore, the study seeks to explore the correlation between troponin-T levels and the development of complications over a four-week timeframe.

## Materials and methods

Type of study

This is a prospective cohort study, and it is also a type of prognostic study.

Sample size

\[ n = \frac{Z^2 \times P \times Q}{d^2} = \frac{(1.96)^2 \times 4 \times 96}{5^2} = 59.006 \]

Where Z is constant (1.96); P is prevalence=4; Q is 100 - P; and d is the error of margin (5%). Hence, we enrolled a total of 60 cases.

Selection of patients

Inclusion Criteria

Patients diagnosed to have acute myocardial infarction as per American College of Cardiology (ACC)/American Heart Association (AHA)/European Society of Cardiology (ESC) guidelines and patients with symptoms of acute myocardial infarction within six hours of onset were included.

Exclusion Criteria

Patients with myopericarditis, known cases of heart failure, pulmonary embolus, sepsis, cardiac contusion, cardiac surgery, and renal disease were excluded.

Method

The diagnosis was based on ECG, biomarkers, and both. Investigations such as troponin T, complete blood picture (CBP) with erythrocyte sedimentation rate (ESR), urea, creatinine, chest X-ray posteroanterior (PA) view, and electrocardiogram (ECG) were assessed. Transthoracic echocardiography was performed at admission to assess for the presence or absence of regional wall motion abnormality (RWMA). Venous blood sample was taken from study participants at the time of admission for cTnT measurement. Cardiac troponin T was measured by using a quantitative immunological test. Outcome included recurrent angina, heart failure, reinfarction, and death, and the study compared the values of troponin T to assess prognosis based on these levels.

Cut-off point of 0.18 ng/mL was taken as mentioned in the kit (Elecsys Troponin T; Cat no. 11621947196, Ref: 11621904193, Roche Diagnostics GmbH, Sandhofer Strasse 116, D-68305 Mannheim). cTnT is considered positive if >0.18 ng/mL and negative if ≤0.18 ng/mL. cTnT levels were divided into tertiles: Lower: ≤0.22 ng/mL; Middle: 0.23-1.17 ng/mL; and Upper: 1.24-4.9 ng/mL.

All patients were observed, and the end points were observed as heart failure, which is defined as left ventricular ejection fraction (LVEF) <40%. If any patient died before follow-up, the LVEF recorded at admission was considered.

Ethics statement

This study was conducted after approval of the Institutional Ethical Committee, Nimra Institute of Medical Sciences (NIMS), Vijayawada, India (IEC/NIMS-VJA/2023-GM.02, Dated: 15-04-2023). The study followed the Declaration of Helsinki, and the subjects provided written informed consent. The current study protocol is approved by the Institutional Ethics Committee, and informed consent was obtained from the attender.

Statistical analysis

Data was analyzed by using software Statistical Package for Social Studies (SPSS 26; IBM Corp., New York, NY, USA). Qualitative data was represented as frequencies and percentages and analyzed using either the Chi-square test or Fisher's exact test. Continuous variables were summarized as mean ± standard deviation. The prognostic value of troponin-T in predicting severity, complications, and outcomes was assessed through logistic regression and receiver operating characteristic (ROC) curves. A p-value of 0.05 or lower was deemed statistically significant. Diagnostic measures, including sensitivity, specificity, positive predictive value (PPV), negative predictive value (NPV), and accuracy, were calculated to evaluate the correlation between troponin-T prognostic levels and outcomes. Kaplan-Meier cumulative mortality curves were constructed, differentiated by the presence or absence of acute myocardial infarction (AMI) and admission cTnT values above or below the detection threshold of 0.18 ng/mL, with group comparisons conducted using the log-rank test.

## Results

Troponin T was positive in 50 (83.34%) and negative in 10 (16.66%) cases. Forty-five patients were male (75%), and 15 were female (25%). The mean age was 53.94±8.672 years for those who tested for cTnT. In 60 patients, 26 (43.34%) patients had anterior wall myocardial infarction alone and eight (13.34%) had inferior wall myocardial infarction alone (Table 1). The remaining 26 (43.34%) had combinations of anterior, inferior, posterior, and right ventricular myocardial infarction.

**Table 1:** Basic characteristics and their distribution in cardiac troponin-T levels

		cTnT Level		Total
		Negative	Positive	
Sex	Female	3	12	15
	Male	7	38	45
Current smoker	No	9	32	41
	Yes	1	18	19
Hypertension	No	5	15	20
	Yes	5	35	40
Diabetes mellitus	No	5	20	25
	Yes	5	30	35
Dyslipidemia	No	4	4	8
	Yes	6	46	52
Mortality	No	10	37	47
	Yes	0	13	13
Killip	I	9	9	9
	II	24	24	24
	III	33	33	33
ST-segment myocardial infarction	No	10	10	10
	Yes	35	35	35
Q-wave myocardial infarction	No	6	6	6
	Yes	27	27	27
Anterior M	No	4	4	4
	Yes	36	36	36
Diastolic dysfunction	I	8	8	8
	II	14	14	14
	III	22	22	22
Chest pain	No	5	16	21
	Yes	5	34	39
Palpitations	No	5	30	35
	Yes	5	20	25
Reperfusion or antiplatelet therapy	No	0	0	0
	Yes	10	50	60
Reperfusion/revascularization	No	10	20	30
	Yes	0	30	30
Recurrent angina	No	10	31	41
	Yes	0	19	19
Heart failure	No	10	32	42
	Yes	0	18	18
Total		10	50	60

Among the 60 patients, 43.34% had anterior wall myocardial infarction (AWMI) and the remaining 56% had non-anterior wall myocardial infarction (AWMI). The number of patients with diabetes, hypertension, and dyslipidemia was 35 (58.34%), 40 (66.67%), and 52 (86.67%), respectively. About 19 (31.67%) patients gave a history of smoking. A significant association was observed between cTnT level and dyslipidemia (p=0.007). Twenty percent of cases belonged to Killip class III, 25% to Killip class II, and 55% to Killip class I. There was a significant association observed between cTnT level and Killip classification (p=0.046).

Irregular rhythms were observed in 21 cases, whereas ST-segment myocardial infarction (MI) was recorded in 14 cases (p=0.069). A significant relationship was identified between ST-segment MI and cTnT levels (p=0.046). Q-wave MI occurred in 26 cases, but no significant association was found between Q-wave MI and cTnT levels. Anterior MI was noted in 20 cases, and a significant association was established between anterior MI and cTnT levels (p=0.05). Diastolic dysfunction grade III was observed in 12 cases (20%), and grade II diastolic dysfunction was observed in 26 cases (43.3%). A significant association was observed between diastolic dysfunction grading and T level (p=0.007) (Table 2).

**Table 2:** Independent samples t-test analysis of basic parameters *cTnT levels >0.18 ng/mL=present, cTnT levels <0.18 ng/mL=absent. cTnT: cardiac troponin T.

	*cTnT Level	Mean	Standard Deviation	Standard Error Mean	P Value
Age (years)	Absent	50.70	7.931	2.508	0.520
	Present	53.94	8.672	1.226	
Ejection fraction (%)	Absent	51.00	2.494	0.789	0.0018
	Present	43.36	6.568	0.929	
Body mass index (kg/m^2^)	Absent	27.30	3.683	1.165	0.906
	Present	27.76	3.543	0.501	
Random blood sugar (mg/dL)	Absent	154.50	50.827	16.073	0.850
	Present	146.46	45.005	6.365	
Systolic blood pressure (mmHg)	Absent	142.00	12.065	3.815	0.489
	Present	139.06	14.081	1.991	
Diastolic blood pressure (mmHg)	Absent	90.00	6.667	2.108	0.886
	Present	88.64	6.262	0.886	
Total cholesterol (mg/dL)	Absent	178.10	28.400	8.981	0.036
	Present	214.04	21.191	2.997	
High-density lipoprotein cholesterol (mg/dL)	Absent	43.80	3.425	1.083	0.492
	Present	38.54	4.816	0.681	
Low-density lipoprotein cholesterol (mg/dL)	Absent	107.00	17.981	5.686	0.776
	Present	120.58	17.479	2.472	
Triglyceride (mg/dL)	Absent	179.60	43.447	13.739	0.796
	Present	225.88	50.243	7.105	
Blood urea (mg/dL)	Absent	18.80	2.201	0.696	0.041
	Present	22.62	8.935	1.264	
Hemoglobin (%)	Absent	12.360	1.5911	0.5031	0.078
	Present	10.138	2.5559	0.3615	
Serum creatinine (mg/dL)	Absent	0.9480	0.15754	0.04982	0.127
	Present	1.0088	0.23379	0.03306	
Sodium (mmol/L)	Absent	134.00	4.346	1.374	0.661
	Present	136.08	5.405	0.764	
Potassium (mmol/L)	Absent	4.3100	0.57822	0.18285	0.864
	Present	4.1016	0.62485	0.08837	
Chloride (mEq/L)	Absent	103.80	2.486	0.786	0.005
	Present	101.94	5.060	0.716	
Respiratory rate (breaths per minute)	Absent	26.00	9.404	2.974	0.036
	Present	24.40	7.074	1.000	
Heart rate (breaths per minute)	Absent	105.60	16.939	5.357	0.2052
	Present	84.70	21.908	3.098	

Chest pain was observed in 65.0%, dyspnea in 61.7%, palpitations in 41.7%, sweating in 48.3%, nausea/vomiting in 26.7%, giddiness in 61.7%, and sudden collapse in 30%. Syncope was observed in 28.3% of cases, and a significant association was found between syncope and cTnT levels (p=0.029). Jugular venous pulse (JVP) wave was observed in 38.3% of cases, and a significant association was found between JVP and cTnT levels (p=0.044). Ejection fraction of ≤40% was recorded in 16 cases. There was a significant association noted between % ejection fraction and cTnT levels (p=0.03). Independent samples t-test shows that no significant change was observed between cTnT level and demographics, laboratory measures, and blood pressure parameters. The mean ejection fraction was 43.36±6.568% in the cTnT-positive group compared with 51.00±2.494% in the cTnT-negative group, with a significant difference (p=0.018). The mean cTnT levels were significantly higher in patients who died than survived (2.9207±1.43 vs 0.705±0.634 ng/mL). The mean cTnT level in patients with reinfarction was 1.94±1.429 ng/mL, compared with 0.8627±1.034 ng/mL in patients without reinfarction. The mean cTnT level was 1.9705±1.53 ng/mL in recurrent angina and 0.8217±0.915 ng/mL in patients without recurrent angina (Table 3).

**Table 3:** ANOVA one-way analysis of basic characteristics *cTnT_Tertile: Lower: ≤0.22 ng/mL; Middle: 0.23-1.17ng/mL; and Upper: 1.24-4.9 ng/mL.

	*cTnT Tertile	Mean	SD	95% CI for Mean		Minimum	Maximum	P Value
				Lower	Upper			
Age (years)	Lower	52.06	8.440	47.86	56.25	40	70	0.532
	Middle	55.16	9.471	50.59	59.72	38	72	
	Upper	53.00	8.028	49.53	56.47	40	70	
	Total	53.40	8.575	51.18	55.62	38	72	
Ejection fraction (%)	Lower	49.67	3.049	48.15	51.18	44	55	<0.001
	Middle	46.21	4.995	43.80	48.62	35	56	
	Upper	39.39	6.444	36.60	42.18	28	52	
	Total	44.63	6.709	42.90	46.37	28	56	
Estimated glomerular filtration rate (mL/min/1.73m²)	Lower	66.22	3.766	64.35	68.10	60	72	<0.001
	Middle	61.21	4.328	59.12	63.30	55	68	
	Upper	57.96	4.724	55.91	60.00	50	65	
	Total	61.47	5.463	60.06	62.88	50	72	
Body mass index (kg/m^2^)	Lower	26.83	3.915	24.89	28.78	20	33	0.103
	Middle	27.00	3.037	25.54	28.46	20	32	
	Upper	28.91	3.410	27.44	30.39	24	36	
	Total	27.68	3.539	26.77	28.60	20	36	
Random blood sugar (mg/dL)	Lower	147.94	48.718	123.72	172.17	85	260	0.435
	Middle	137.63	51.499	112.81	162.45	85	280	
	Upper	156.09	37.782	139.75	172.42	85	220	
	Total	147.80	45.666	136.00	159.60	85	280	
Systolic blood pressure (mmHg)	Lower	142.50	14.577	135.25	149.75	125	170	0.493
	Middle	137.11	13.366	130.66	143.55	115	160	
	Upper	139.26	13.478	133.43	145.09	115	160	
	Total	139.55	13.714	136.01	143.09	115	170	
Diastolic blood pressure (mmHg)	Lower	89.44	6.836	86.04	92.84	80	105	0.593
	Middle	87.63	6.743	84.38	90.88	75	100	
	Upper	89.43	5.566	87.03	91.84	80	100	
	Total	88.87	6.294	87.24	90.49	75	105	
Total cholesterol (mg/dL)	Lower	192.00	29.470	177.35	206.65	140	223	0.002
	Middle	209.63	21.014	199.50	219.76	150	250	
	Upper	219.30	21.016	210.22	228.39	185	260	
	Total	208.05	26.047	201.32	214.78	140	260	
High-density lipoprotein cholesterol (mg/dL)	Lower	41.94	4.696	39.61	44.28	32	48	<0.001
	Middle	40.37	4.336	38.28	42.46	33	50	
	Upper	36.65	4.529	34.69	38.61	30	52	
	Total	39.42	4.996	38.13	40.71	30	52	
Low-density lipoprotein cholesterol (mg/dL)	Lower	113.83	20.345	103.72	123.95	80	145	0.149
	Middle	115.63	15.731	108.05	123.21	80	138	
	Upper	124.04	17.416	116.51	131.57	86	152	
	Total	118.32	18.141	113.63	123.00	80	152	
Triglyceride (mg/dL)	Lower	205.22	64.859	172.97	237.48	120	320	0.082
	Middle	207.58	36.459	190.01	225.15	120	254	
	Upper	237.04	47.552	216.48	257.61	165	350	
	Total	218.17	51.836	204.78	231.56	120	350	
Urea (mg/dL)	Lower	18.83	1.855	17.91	19.76	16	24	0.005
	Middle	19.74	3.885	17.86	21.61	12	30	
	Upper	26.30	11.761	21.22	31.39	16	55	
	Total	21.98	8.313	19.84	24.13	12	55	
Hemoglobin (%)	Lower	11.672	2.2418	10.557	12.787	7.0	16.0	0.061
	Middle	9.837	2.3272	8.715	10.959	5.8	12.9	
	Upper	10.152	2.7406	8.967	11.337	5.8	13.5	
	Total	10.508	2.5513	9.849	11.167	5.8	16.0	
Creatinine (mg/dL)	Lower	0.8994	0.14965	0.8250	0.9739	0.73	1.30	<0.0001
	Middle	0.9042	0.14393	0.8348	0.9736	0.73	1.20	
	Upper	1.1543	0.24070	1.0503	1.2584	0.80	1.80	
	Total	0.9987	0.22293	0.9411	1.0563	0.73	1.80	
Sodium (mmol/L)	Lower	136.28	4.897	133.84	138.71	125	146	0.614
	Middle	134.74	4.665	132.49	136.99	126	144	
	Upper	136.13	6.070	133.51	138.76	114	144	
	Total	135.73	5.269	134.37	137.09	114	146	
Potassium (mmol/L)	Lower	4.0833	.69473	3.7379	4.4288	2.70	5.50	0.135
	Middle	4.3637	.64365	4.0535	4.6739	3.00	5.50	
	Upper	3.9900	.49153	3.7774	4.2026	3.10	5.31	
	Total	4.1363	.61757	3.9768	4.2959	2.70	5.50	
Chloride (mEq/L)	Lower	103.17	2.975	101.69	104.65	98	107	<0.0001
	Middle	105.32	3.902	103.43	107.20	98	115	
	Upper	99.00	4.661	96.98	101.02	90	107	
	Total	102.25	4.764	101.02	103.48	90	115	
Respiratory rate (breaths per minute)	Lower	26.78	7.666	22.97	30.59	16	38	0.036
	Middle	26.42	8.023	22.55	30.29	16	38	
	Upper	21.57	5.845	19.04	24.09	14	38	
	Total	24.67	7.444	22.74	26.59	14	38	
Heart rate (breaths per minute)	Lower	101.56	16.825	93.19	109.92	70	140	<0.0001
	Middle	92.42	15.632	84.89	99.96	60	120	
	Upper	74.22	23.821	63.92	84.52	45	126	
	Total	88.18	22.452	82.38	93.98	45	140	

Mortality was found in 13 (21.7%) patients. The mean cTnT was 2.9207±1.43 ng/mL, and the mean ejection fraction (EF) was 35.62±6.06% in patients who died. A comparison of mean cTnT between patients with and without mortality was done, and the difference was found significant with cTnT (2.9207±1.43 vs 0.705±0.634 ng/mL, p<0.0001). Recurrent angina was found in 19 (31.67%) patients. The mean cTnT was 1.9705±1.538 ng/mL, and the mean EF was 39.947±7.1295% in patients with recurrent angina. A comparison of mean cTnT between patients with and without recurrent angina was done, and the difference was found significant with cTnT (1.9705±1.538 vs 0.82±0.915 ng/mL, p<0.0001).

A total of 18 (30%) patients had heart failure. The mean cTnT was 2.34±1.559 ng/mL, and the mean EF was 38.22±6.8389% in patients with heart failure. Thirty-six percent (18/50) of the TnT-positive cases have heart failure. About 66.67% (12/18) of the TnT-positive cases have died due to heart failure. About 57.9% (11/19) of the cTnT-positive cases with recurrent angina died. For diagnosis of AMI, a single cut-off value for cTnT (0.22 ng/mL) at presentation resulted in a sensitivity and negative predictive value of 76.92% (95% CI: 88.4%-95.3%) and 98.2% (95% CI: 96.5%-98.5%), and a specificity and positive predictive value of 100% (95% CI: 92.3%-100%) and 65.4% (95% CI: 48.3% 75.3%), respectively. For diagnosing AMI with mortality, a cut-off value for cTnT (2.2 ng/mL) resulted with a sensitivity and negative predictive value of 76.923% (95% CI: 46.187%-94.962%) and 94.000% (95% CI: 85.309%-97.689%), and a specificity and positive predictive value of 100% (95% CI: 92.451%-100%) and 100% (95% CI: 93.55%-100%), respectively. The admission cTnT levels >2.2 ng/mL were used to predict mortality in AMI patients with an area under the ROC curve (AUROC) of 0.885.

The ROC curve analysis reveals an area under the curve (AUC) of 0.739 for cTnT levels in predicting patients with recurrent angina with a sensitivity of 47.37% and a specificity of 97.56%, with an AUC of 0.739 (95% CI: 0.609-0.844). The admission cTnT levels above 1.9 ng/mL were used to predict heart failure in AMI patients with an AUROC of 0.702 (Table 4).

**Table 4:** Cardiac troponin T (cTnT) cut-off to predict mortality *Mortality: Yes= died, No=survived.

cTnT (ng/mL)	*Yes	No	Total
>2.2 ng/mL	10	0	10
≤2.2 ng/mL	03	47	50
Total	13	47	60

**Figure 1:**
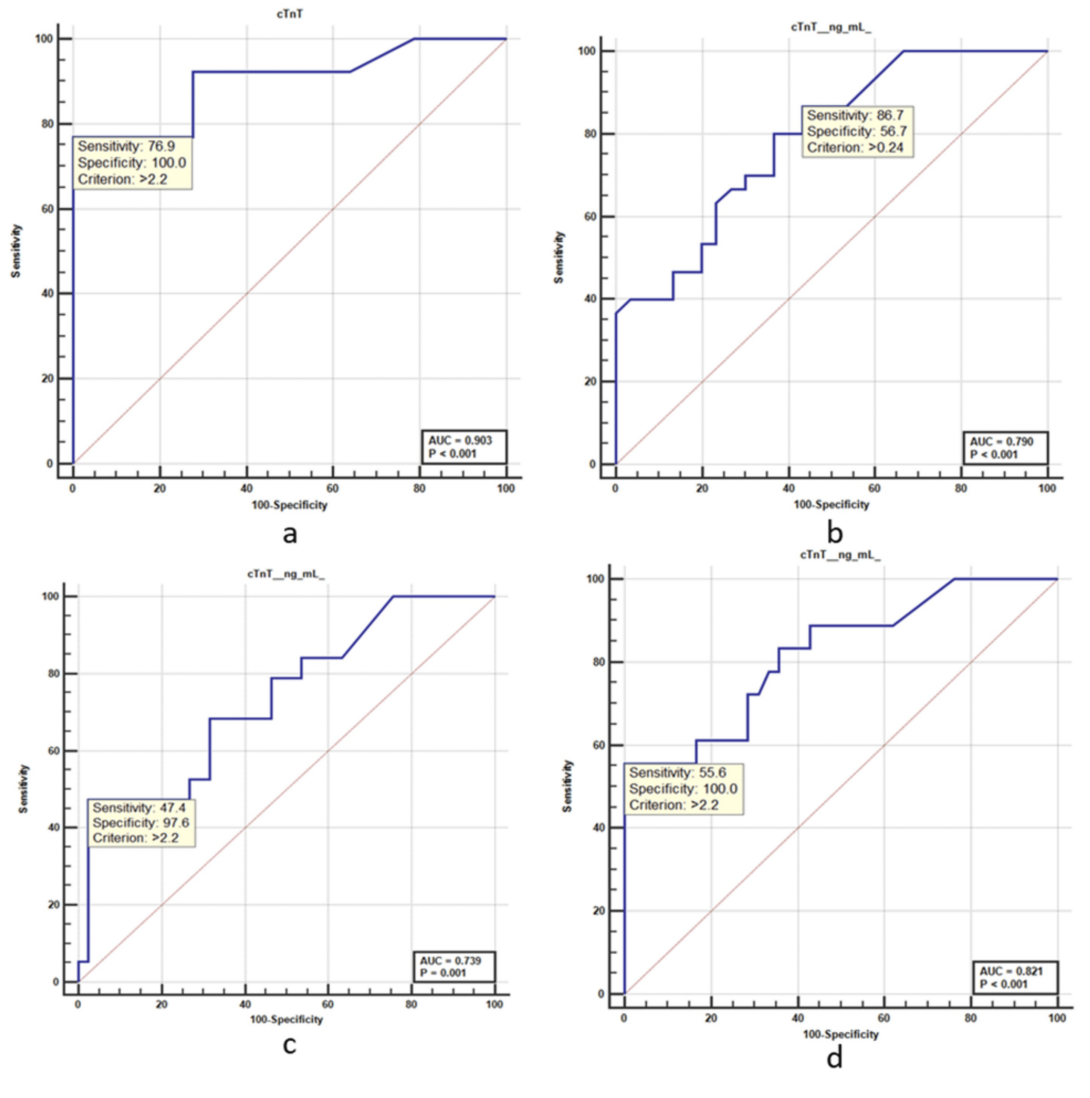
Receiver operating characteristic (ROC) curve a. Receiver operating characteristic (ROC) for outcome (mortality). b. ROC curve for reperfusion/revascularization. c. ROC curve for recurrent angina. d. ROC curve for heart failure. cTnT: cardiac troponin T.

Whereas for diagnosing AMI with revascularization, a cut-off value for cTnT (0.24 ng/mL) resulted with a sensitivity and negative predictive value of 86.667% (95% CI: 69.278%-96.245%) and 80.952% (95% CI: 61.832%-91.769%), and a specificity and PPV of 56.667% (95% CI: 37.427%-74.539%) and 66.667% (95% CI: 56.477%-75.506%), respectively. The admission cTnT levels above 0.24 ng/mL were used to predict revascularization in AMI patients with an AUROC of 0.717. The ROC curve analysis reveals an AUC of 0.790 for cTnT levels in predicting patients with revascularization, with a sensitivity of 86.67% and a specificity of 56.67%, with an AUROC of 0.790 (95% CI: 0.665-0.884). Multivariable analysis showed the strongest association of mortality with cardiac troponin T ≥0.22 ng/mL (HR: 1.95, 95% CI: 1.85-2.19), heart failure (HR: 1.85, 95% CI: 1.65-2.08), recurrent anginal (HR: 1.38, 95% CI: 1.19-1.55), and reinfarction (HR: 1.04, 95% CI: 0.99-1.12). Patients with cardiac troponin T ≥2.2 ng/ml have a poor prognosis irrespective of the final diagnosis (Figure 1).

## Discussion

The increasing incidence of acute myocardial infarction (AMI) in India is associated with changes in lifestyle and various risk factors [18]. A major obstacle in India is the restricted availability of coronary angiography in many facilities that treat STEMI patients [19]. Among the newer indicators of myocardial injury, troponin T (TnT) is particularly specific to cardiac tissue and is released into the bloodstream slightly earlier than creatine kinase (CK) in cases of myocardial damage. Research has shown that elevated cardiac troponin-T (cTnT) levels upon admission are associated with a heightened risk of adverse cardiac events in patients suffering from acute ST-segment elevation myocardial infarction (STEMI). The reasons behind the prognostic importance of elevated cTnT at the time of admission are not fully understood; however, it may relate to more extensive myocardial injury in patients who arrive later after the onset of symptoms, a higher incidence of recanalization failure, and less effective microvascular perfusion.

The present study examined 60 patients diagnosed with acute myocardial infarction (AMI) who were admitted to the intensive care unit (ICU). In this study, 45 patients were male (75%) and 15 were female (25%). There is no significant relationship between sex and cTnT levels (p=0.689), suggesting that sex does not affect cTnT expression in patients with AMI. TnT positivity was correlated with the type of acute myocardial infarction and associated complications. Out of 10 TnT-negative patients with complications, two cases had AWMI. In 50 TnT-positive patients with complications, 43.34% had AWMI. Thus, an increase in complications was found in TnT-positive AWMI group (43.34% vs 10%). This was statistically significant.

A significant association was observed between cTnT level and dyslipidemia (p=0.007). The occurrence of complications was higher in the cTnT-positive group. A significant relationship was identified between ST-segment MI and cTnT levels (p=0.046). Reperfusion or antiplatelet therapy was performed in 83.3% of cases, whereas reperfusion/revascularization was performed in 60% of cases. There was a significant association observed between recurrent angina and cTnT level (p=0.018) and heart failure and cTnT level (p=0.023). Although the exact mechanisms are not yet fully elucidated, evidence suggests that patients with elevated troponin levels, especially those with non-ST-segment elevation MI, exhibit a higher incidence of coronary thrombi, more complex lesions, and impaired coronary flow.

Moreover, research has demonstrated a relationship between cTnT levels measured 72 hours after MI and infarct size, regardless of reperfusion status [18,19]. The mean cTnT levels were higher in patients with heart failure than without heart failure. Among the total cases of acute myocardial infarction, 30 patients underwent thrombolytic therapy utilizing acyl plasminogen streptokinase activated complex, whereas 20 patients were treated with streptokinase. A receiver operating characteristic (ROC) curve was generated using all cTnT data obtained at admission, with samples collected within one hour of the onset of chest symptoms. The diagnostic performance of cTnT was evaluated through sensitivity and specificity analysis based on the determined cut-off value. A comparison of mean cTnT between patients with and without mortality was done, and the difference was found significant with cTnT (p<0.001). Twenty-six percent (13/50) of the cTnT-positive cases have died. Stubbs et al. showed that a positive troponin T (TnT) result at the time of admission was associated with a higher probability of experiencing future cardiac events and mortality during the follow-up period, with rates of 15% compared to 5% at one year and 28% vs 7.5% at three years [20].

Recurrent angina was found in 19 (31.67%) patients. The mean cTnT was 1.97±1.53 ng/mL, and the mean EF was 39.94±7.12% in patients with recurrent angina. A comparison of mean cTnT between patients with and without recurrent angina was done, and the difference was found significant with cTnT (1.9705±1.538 vs 0.82±0.915 ng/mL, p<0.001). Totally, 38% (19/50) of the TnT-positive cases have recurrent angina. Thirty-six percent (18/50) of the TnT-positive cases have heart failure. About 66.67% (12/18) of the TnT-positive cases have died due to heart failure. A study by Kazmi et al. evaluated the relationship between admission cTnT response to streptokinase in acute myocardial infarction [21]. The exact mechanism that connects elevated levels of cardiac troponin T (cTnT) at the time of admission to an increased risk of mortality is still not fully understood. One possible reason for the poorer prognosis seen in patients with positive cTnT results is the prolonged interval between the onset of symptoms and the commencement of reperfusion therapy.

The ROC curve analysis reveals an area under the curve (AUC) of 0.903 for cTnT levels in predicting chest pain patients with a confirmed case of AMI (cTnT >0.22). The cut-off of cTnT with 0.22 ng/mL at admission was found to be the predictor of patients with a confirmed case of AMI with a sensitivity of 76.92% and a specificity of 100%, with an area under the ROC curve (AUROC) of 0.903 (95% CI: 0.798-0.964). Although the achieved negative predictive value is extremely high, it is important to stress that the cTnT one-hour algorithm should always be used along with full clinical assessment, including patient examination, and 12-lead ECG. Positive predictive value for acute MI in the upper zone cTnT was 79.5%, with a sensitivity of 76.92% and a specificity of 100.00%.

For diagnosing AMI with recurrent angina, a cut-off value for cTnT (2.2 ng/mL) resulted with a sensitivity and NPV of 47.368% (95% CI: 24.447%-71.136%) and 80% (95% CI: 72.25%-86.004%), and a specificity and PPV of 97.561% (95% CI: 87.145%-99.938%) and 90% (95% CI: 55.085%-98.50%), respectively. The admission cTnT levels above 2.2 ng/mL were used to predict heart failure in AMI patients with an AUROC of 0.778. The ROC curve analysis reveals an area under the curve (AUC) of 0.821 for cTnT levels in predicting patients with heart failure, with a sensitivity of 55.56% and a specificity of 100%, with an area under the ROC curve (AUROC) of 0.821 (95% CI: 0.700-0.908). The ROC curve analysis reveals an AUC of 0.756 for cTnT levels in predicting patients with reinfarction, with a sensitivity of 50% and a specificity of 90.48%, with an area under the ROC curve (AUROC) of 0.756 (95% CI: 0.628-0.858).

Our results show that a troponin T level exceeding 2.6 ng/mL was predictive of a left ventricular ejection fraction (LVEF) <40%. The cut-off value of 0.22 ng/mL exceeds the limit set by manufacturers, who claim that any cardiac troponin T >0.18 ng/mL indicates myocardial injury. Previous study employed thresholds of 0.5 µg/L and 1.0 µg/L, while a subsequent multi-center study by the same research team established a cut-off of 0.2 µg/L [22]. Furthermore, another research group identified an upper reference limit of 0.5 µg/L [23]. This evidence supports the validity of a 0.2 µg/L threshold, suggesting that a cut-off of 0.5 µg/L is excessively high.

A study by Ohman et al. was conducted in 855 patients within 12 hours of the onset of symptoms [24]. They evaluated cardiac troponin-T levels, creatine kinase-myocardial band (CK-MB) levels, and electrocardiograms. Among the 801 patients with baseline serum samples, 289 showed elevated troponin T levels (>0.1 ng/mL) at the time of admission. The 30-day mortality rate was significantly higher in these patients compared to those with lower troponin T levels, with rates of 11.8% vs 3.9% (p< 0.001). In their study, troponin T levels remained a significant predictor of 30-day mortality even after adjusting for electrocardiographic categories and CK-MB levels (p=0.027) [24].

Another study by Rasmussen et al. assessed the predictive ability of routine prehospital point-of-care cardiac troponin T in the risk stratification of patients suspected of having acute myocardial infarction (AMI) [25]. A diagnosis of AMI was confirmed in 2,187 cases, of which 2,150 point-of-care cardiac troponin T measurements (11.0%) indicated levels of ≥50 ng/mL, including 966 cases of AMI, resulting in a sensitivity of 44.2% and a specificity of 92.8%. Patients with a prehospital cardiac troponin T level of ≥50 ng/mL experienced a one-year mortality rate of 24%, compared to 4.8% for those with levels below 50 ng/mL. The multivariable analysis demonstrated that several factors significantly associated with mortality were cardiac troponin T ≥50 ng/mL (HR: 2.10, 95% CI: 1.90-2.33), congestive heart failure (HR: 1.93, 95% CI: 1.74-2.14), diabetes mellitus (HR: 1.42, 95% CI: 1.27-1.59), and increasing age (HR: 1.08, 95% CI: 1.08-1.09). Importantly, patients with cardiac troponin T levels of ≥50 ng/mL demonstrated a poor prognosis, irrespective of the final diagnosis [25].

A study by Reichlin et al. validated a novel one-hour algorithm that employs high-sensitivity cardiac troponin T measurements for accurate diagnosis of acute myocardial infarction [26]. Acute MI was confirmed in 17.3% of the patients. Using the high-sensitivity cardiac troponin T one-hour algorithm, 786 patients (59.5%) were classified as "rule-out," 216 patients (16.4%) as "rule-in," and 318 patients (24.1%) as falling within the "observational zone." The sensitivity and negative predictive value for acute MI in the rule-out category were determined to be 99.6% (95% CI: 97.6%-99.9%) and 99.9% (95% CI: 99.3%-100%). In the rule-in category, the specificity and positive predictive value for acute MI were 95.7% (95% CI: 94.3%-96.8%) and 78.2% (95% CI: 72.1%-83.6%) [26].

Our results indicate that while an increase in cTnT correlates with a worse short-term prognosis, troponin T is a more reliable prognostic marker when arbitrary cut-off values are not utilized in assessments. Our findings support the notion that this lower troponin T threshold is beneficial for diagnosing infarction and for identifying patients at increased risk of mortality and morbidity. This study concludes that the presence of cTnT in the bloodstream is a sensitive and specific marker of myocyte injury, with a diagnostic cut-off set at 0.22 ng/mL. When used to confirm myocardial infarction in patients presenting with chest pain, this marker is most effective when measured at any time within 24 hours of admission. Additionally, this research demonstrates that the cardiac troponin T level measured immediately upon admission serves as a strong, independent risk marker for patients experiencing acute myocardial infarction. The combined application of electrocardiographic criteria and cardiac troponin T levels may improve the early management of these patients.

Limitations

The study was conducted with a limited number of patients, which requires careful consideration when interpreting the results. Cardiac troponin-T (cTnT) levels were measured only at the time of admission to know the short-term prognosis, with no follow-up assessments conducted thereafter. We stated the limitation that further studies with a larger, multicenter patient population are needed. Various studies have investigated the causes of elevated troponin T beyond acute myocardial infarction (MI), confirming that chronic kidney disease (CKD), pulmonary embolism (PE), and myocarditis are among the most common non-ischemic causes. Hence, it should be restricted during the enrolment of patients.

## Conclusions

Elevated cTnT levels can assist in identifying high-risk patients who may benefit from close monitoring and the timely management to improve the outcome. The routine assessment of troponin in prehospital settings has proven to be highly predictive, facilitating the identification of high-risk patients before hospital admission and enabling their direct transfer to specialized care facilities. Cardiac troponin T acts as a strong, independent risk factor for patients with acute myocardial infarction, thereby enhancing risk stratification when combined with echocardiography. Cardiac troponin-T levels at admission are accurate to predict the short-term prognosis, mortality, recurrent ischemic events, and heart failure in patients with acute myocardial infarction.
